# “[I]t will be like a full course meal”: healthcare worker perspectives on strategies to optimize integration of PrEP into medication for opioid use disorder and needle and syringe exchange programs for people who use drugs in Kampala, Uganda

**DOI:** 10.1186/s13722-026-00693-z

**Published:** 2026-06-27

**Authors:** Nok Chhun, Brenda Kamusiime, Alisaati Nalumansi, Chris Collins Twesige, Grace Kakoola Nalukwago, Vicent Kasiita, Peter Mudiope, Ritah Kansiime, Timothy R. Muwonge, Peter Kyambadde, Herbert Kadama, Sara Glick, Barrot Lambdin, Renee Heffron, Andrew Mujugira, Kristin Beima-Sofie

**Affiliations:** 1 Department of Global Health, The University of Washington, 3980 15th Ave NE, Seattle, WA 98195 USA; 2 Infectious Diseases Institute, Makerere University, Kampala, Uganda; 3 Ministry of Health, Kampala, Uganda; 4 Most-At-Risk Populations Initiative (MARPI), National STI Control Unit, Kampala, Uganda; 5 Division of Allergy and Infectious Diseases, Department of Medicine, University of Washington, Seattle, WA USA; 6 Research Triangle International, San Francisco, CA USA; 7 Department of Medicine, University of Alabama at Birmingham, Birmingham, AL USA

**Keywords:** PrEP, People who use drugs, Harm reduction, Consolidated framework for implementation research (CFIR), Healthcare worker, Uganda

## Abstract

**Background:**

Integrating pre-exposure prophylaxis (PrEP) into facility-based medication for opioid use disorder (MOUD) and community-based needle and syringe exchange (NSP) programs may optimize service provision and reduce HIV acquisition among people who use drugs (PWUD). Healthcare workers (HCWs) providing PrEP or substance use services to PWUD offer important insight into potential challenges and strategies for effective integration.

**Methods:**

Between March 2021—September 2022 HCWs experienced in PrEP or substance use service provision for PWUD were purposively sampled from five program sites in Kampala, Uganda that offered MOUD, NSP or in-patient and outpatient rehabilitation services. Interviews were guided by the Consolidated Framework for Implementation Research and conducted with a range of HCW cadres, including nurses and social workers. Sample size was determined based on information power. Interviews were conducted by Ugandan social scientists, audio recorded, translated, and transcribed verbatim. Directed content analysis was used to identify HCW perspectives on determinants affecting implementation of and priority strategies for integrating PrEP with MOUD and NSP services.

**Results:**

Thirty HCW interviews were conducted; the median age of participants was 32 years (IQR: 28–36), and 54% identified as male. HCW knowledge about PrEP varied across program sites, but all recognized the importance of HIV prevention among PWUD and advocated for training to ensure successful integration of PrEP with MOUD and NSP service programs. By offering the “whole package” of services in one setting, HCWs felt that PrEP service integration prioritized client needs and provided a relative advantage when compared to existing facility-based delivery. HCWs were most enthusiastic about community-based PrEP integration strategies that would take “services to clients where they are,” address client concerns about stigma experienced in facilities, and remove prohibitive transportation costs. HCWs recommended community-based refill models and longer-acting PrEP products to address transportation barriers, engaging peers in delivery approaches to reduce stigma and create “friendly” environments that improve service utilization and having a dedicated PrEP implementation “champion” to optimize integration.

**Conclusions:**

HCWs viewed PrEP integration with MOUD and NSP services as acceptable, appropriate, and feasible and recommended peer delivery as a best approach, especially in community settings, such as at drop-in centers focusing on service provision for PWUD.

**Supplementary Information:**

The online version contains supplementary material available at 10.1186/s13722-026-00693-z.

## Introduction

The United Nations Office on Drugs and Crime reported that in 2022, 292 million people worldwide used drugs for non-medical purposes, and among them, 13.9 million reported injecting drugs [1]. People who use drugs (PWUD), including those who inject, need access to HIV treatment and prevention services to limit HIV transmission. Globally, among those who inject drugs, the risk for HIV is 14 times higher than in the general population [1]. The Uganda Population-Based HIV Impact Assessment reported a national HIV prevalence of 5.9% among adults aged 15–64 years, with 6.0% prevalence reported for Kampala, Uganda [2]. Although available data are limited, among PWUD who also engage in transactional sex, an estimated 31.3% are living with HIV [3]. Among people who inject drugs (PWID), that estimate ranges from 3.6% to 45% [4–9], depending on population, data collection methods and procedures.

Medication for opioid use disorder (MOUD) and needle and syringe exchange (NSP) programs reduce the risk of overdose and HIV transmission among PWUD. Integration of MOUD services with HIV treatment has been shown to improve anti-retroviral therapy (ART) initiation rates for PWID [10] as well as ART adherence [11] and viral suppression [11, 12]. In Tanzania, having access to HIV treatment within an opioid treatment program increased 90-day ART initiation by two-fold (41% to 87%; *p* < 0.001) [10]. Furthermore, delivery of directly observed ART (DOT) at methadone maintenance clinics in the Bronx, New York, USA, improved both ART adherence [86% in the DOT group compared to 56% in the control group (*p* < 0.0001)] and viral suppression (DOT participants with undetectable VL increased from 51% to 71%) [11]. Additionally, in Kazakhstan, an integrated service delivery model that combined NSP with HIV services found significant increases in HIV testing, higher rates of ART initiation, and improved viral suppression [13]. However, few programs have integrated pre-exposure prophylaxis (PrEP) with MOUD and NSP programs. In Uganda, PrEP was implemented in HIV treatment clinics following the release of national guidance in 2016 [14] in alignment with the World Health Organization’s 2015 HIV prevention guidelines [15], and has since expanded to additional service settings, including family planning and STI clinics [16]. Optimizing PrEP delivery within MOUD and NSP service settings for people who use or inject drugs may reduce HIV acquisition in these underserved populations.

Healthcare workers (HCW) have played a pivotal role in HIV prevention by promoting the use and delivery of PrEP specifically among adolescent girls and young women (AGYW), a priority population [17–20]. When HCWs approach HIV counseling and PrEP delivery in a non-judgmental and stigma-free manner, they have been instrumental in facilitating PrEP uptake [21]. PrEP remains a relatively newer HIV prevention strategy in many settings, therefore, understanding provider perspectives on its integration into existing services is essential [22–24]. Perspectives from HCWs who provide care to PWUD are critical for successful integration of PrEP with MOUD and NSP service programs. Given their role in their organizational settings, HCWs offer valuable insights that can identify key facilitators (e.g., person-centered care), and barriers (e.g., stigma) to service integration. Including HCW perspectives can support development of patient-centered strategies for integration because HCWs are ideally poised to identify gaps in and advocate for policies that support feasibility and acceptability. Specifically, HCWs can leverage current workflows to understand best practices to incorporate HIV PrEP delivery, ensure that integrated services are aligned with client needs and delivered in stigma-free settings, and utilize their existing client relationships to facilitate trust with patients when new services are introduced [22, 25].

Evidence from community-based models (e.g., pharmacy-based and mobile clinic delivery) demonstrated improved PrEP uptake and acceptability across diverse populations [26, 27]. Additionally, peer-led approaches enhanced engagement, reduced concerns around stigma and confidentiality, and improved uptake by delivering services in trusted community settings [28–33]. We conducted a qualitative assessment to inform strategies for optimizing PrEP delivery within existing MOUD and NSP service programs for PWUD in Uganda. Our goal was to identify key determinants to acceptability, appropriateness, and feasibility of PrEP integration. We characterized healthcare workers’ previous experiences delivering substance use and HIV prevention services for PWUD. We also highlighted recommendations for integration and scale-up of PrEP across facility-based MOUD and community-based NSP sites in Kampala, Uganda.

## Methods

### Study design, setting, and population

We conducted a formative qualitative assessment to inform PrEP delivery within existing MOUD and NSP service programs for PWUD in Kampala, Uganda. The purpose of this formative work was to guide an implementation science study focused on PrEP integration with a facility-based MOUD program located within Butabika National Referral Mental Hospital [7, 34] and a community-based NSP program led by the Most-At-Risk Populations Initiative (MARPI), a non-profit organization. Facility-based delivery refers to services that are delivered in fixed, formal health settings, such as in hospitals, clinics, and health centers. This contrasts with community-based- or outreach-based service delivery models utilized in our study which includes both fixed community venues such as drop-in centers and informal areas in the community where PWUD congregate. Partnering programs were selected through consultations with the Ugandan Ministry of Health. At the time of the study, the Butabika National Referral Mental Hospital housed the country’s only MOUD program, established in 2019. MARPI leads the country’s only NSP program, with satellite sites including Hope and Beyond Rehabilitation Center. Additional partners from substance use rehabilitation centers were identified based on their active community engagement and willingness to partner with the Infectious Diseases Institute research team including Serenity Rehabilitation Center. Additional substance use partners and programs exist and are similar in organizational structure but generally offer fewer services and serve fewer clients than those we partnered with for this work.

Study screening and recruitment were conducted in partnership with the five participating substance use program sites, which included MOUD (*n* = 1), NSP (*n* = 2) and in-patient and outpatient rehabilitation centers (*n* = 2). Supervisors and key personnel at the sites disseminated information about the study and referred potentially eligible HCWs to the study team. In Uganda, the Ministry of Health broadly defines the health workforce as any person, regardless of formal training, who contribute to the “protection and improvement of health. This includes all health workers, whether employed in government service, non-governmental organizations, the private sector, whether self-employed or volunteers” [35]. The range of roles represented under this definition include for example, medical doctors, nurses, midwives, social work and counseling professionals, clinical psychologists, pharmacists, clinical officers, community health workers, and health service managers [36]. In our study, HCWs were eligible to participate if they were ≥ 18 years old and provided substance use and/or PrEP/HIV services to PWUD. There was no eligibility criteria related to duration of employment, specific roles, or level of involvement in service delivery. We interviewed HCWs from the program sites to understand their beliefs and experiences about PrEP and substance use services with the goal of optimizing PrEP integration into MOUD and NSP service programs for PWUD. We purposively sampled a range of HCW cadres who provided substance use services to PWUD. In addition to these services, HCWs also offered a range of other services to PWUD, including HIV services, sexual and reproductive health, mental health, substance use counseling, and other social services. Supported by previous literature, and given participant heterogeneity, we anticipated that conducting more than 15 interviews would allow us to achieve sufficient data saturation [37] and provide sufficient information power to characterize participant experiences [38].

### Data collection

The research team conducted semi-structured in-depth interviews with HCWs from March 2021 to September 2022. The Consolidated Framework for Implementation Research (CFIR), a meta-theoretical framework designed to identify implementation determinants, guided data collection, analysis, and evidence interpretation [39, 40]. The CFIR has been previously applied to assess barriers and facilitators to HIV services integration with opioid treatment services for PWUD [41] and syringe service programs with PWID [42]. Qualitative data from in-depth interviews were collected using a topic guide (supplementary file 1) that addressed each of the five CFIR domains (intervention characteristics, inner setting, outer setting, characteristics of individuals, and process). An additional domain, characteristics of systems, along with constructs relevant in low- and middle-income settings, such as resource continuity and community characteristics, were also included [40]. Although the interview guide was not pilot tested prior to data collection, questions were informed and adapted from construct-specific example CFIR questions to align to our study objective [43]. The data collection team included Ugandan social scientists (BK, AN, CCT, GKN, VK) who conducted all in-depth interviews in English or Luganda, depending on HCW preference. The data team administered a demographic survey prior to interviews to collect information on participant characteristics, including sex, age, education, current employment classification, years working at the clinic, years of experience providing HIV care, and years of experience providing care to PWUD. In addition, we assessed their level of confidence in providing MOUD, NSP, or PrEP. Furthermore, we collected information on HCWs’ perceptions regarding the acceptability, appropriateness, and feasibility of integrating PrEP with MOUD and NSP services in a how much do they agree format using a Likert scale from 1 to 5 (1 = strong disagreement; 5 = strong agreement). These questions were adapted from standardized measures assessing acceptability, appropriateness, and feasibility [44] and were used in our formative research to indicate the potential for PrEP integration success. During the interview, HCWs were asked about their role at the program site, experience providing MOUD, NSP and/or HIV prevention services to PWUD, their opinion about PrEP integration into MOUD and NSP programs, and potential challenges and strategies for optimizing PrEP integration. Interviews were audio-recorded, translated if necessary, and transcribed verbatim. Each interview lasted a median of 60 min (interquartile range [IQR]: 52–72).

The research protocol was approved by the Mildmay Uganda Research Ethics Committee (0309–2020), the Uganda National Council for Science and Technology (HS1202ES), and the University of Washington Human Subjects Division (STUDY00010421). All participants provided written informed consent in English or Luganda, the local language. For their time, participants received a reimbursement of 30,000 shillings (8 USD), which is consistent with local ethics regulations.

### Data analysis

Directed content analysis [45] of transcripts identified HCWs’ perspectives on determinants of PrEP integration with MOUD and NSP services. Relevant CFIR constructs within each domain served as an overall guide for developing an initial codebook by four members of the research team (BK, AN, NC, and KBS). Once the initial codebook was developed, a subset of transcripts was coded by all team members to refine code definitions, add additional codes identified inductively from transcripts, and agree on text segmentation practices. Once the codebook was finalized, four coders (BK, AN, NC, and KBS) independently coded assigned transcripts, which were then independently reviewed by another team member to confirm coding agreement. Discrepancies were reviewed until consensus was reached through team discussion. Queries were generated to summarize findings within CFIR domains and constructs. ATLAS.ti software (version 25) was used to support data management and analysis.

Descriptive statistics of HCWs and their experiences with MOUD, NSP, and PrEP, their level of confidence in providing these services, and their perception of the acceptability, appropriateness, and feasibility of PrEP integration with MOUD and NSP services were summarized using counts, proportions, medians, and interquartile ranges. Quantitative analysis was performed using the R statistical computing environment [46].

## Results

Thirty HCWs were interviewed across five participating program sites (Table 1). Two HCWs were excluded from the analysis because one interview was missing an audio file (consequently, there was no interview transcript) and one participant was later found to not meet eligibility criteria. The remaining HCWs (*n* = 28) represented a range of cadres, including social workers (*n* = 8), nurses (*n* = 8), psychologists (*n* = 7), and clinical officers (*n* = 3). The median age of HCWs was 32 years (IQR: 28–36), and 54% identified as male. HCWs reported a median of 3 years (IQR: 1–6) at their facility, a median of 5 years (IQR: 2–6) providing HIV care, and a median of 4 years (IQR: 2–6) of experience providing care to PWUD (Table 2).

**Table 1:** Description of program sites (*N* = 5) providing services to people who use drugs in Kampala, Uganda

Program Site	Description	Patient Volume	Substance Use Services	HIV Services	Pharmacy On-site	Service Delivery Setting
Butabika National Referral Mental Hospital	Medication-assisted treatment (MAT) clinic opened in September 2020	• 550 bed-capacity^1^ • 1,100–1,300 in-patient; 350 out-patient volume^1^	• Methadone/buprenorphine for people with opioid use disorder • Psychosocial support	• HIV testing and counseling • PrEP referral to Makerere University Joint AIDS Program	• Operates 24/7	Facility-based delivery via the MAT clinic
Uganda Harm Reduction Network (UHRN)	Civil Society Organization providing services to people who use drugs (PWUD) or inject drugs (PWID). UHRN operates the drop-in centers (DICs) Established in 2008	• Subscriber base of 4,500 individuals, and over 26 civil society and community-based organizations across 40 districts in Uganda^2^	• Needle and syringe exchange (NSP) program services • Community-based screening and referral to Butabika MAT clinic for individuals who may benefit from medications for opioid use disorder (MOUD) • Psychosocial support • Overdose management using Naloxone	• HIV prevention services including testing and counseling • Referral for ART and PrEP services • Partner with health facilities for PrEP initiation at DICs as part of weekly DIC outreach activities • PrEP refills offered via facility-based delivery or through peers via community-based delivery	• None	Community-based delivery via drop-in centers and outreach activities in informal areas where PWUD congregate
Most-At-Risk Population Initiative (MARPI)	MARPI is affiliated with the sexually transmitted infections control unit Mulago Hospital Since 2008, MARPI has been with key populations	• Has served over 500,000 clients^3^	• NSP services • Collaborate with UHRN and utilize the DIC to provide services • Referral to Butabika MAT clinic for MOUD • Psychosocial support	• HIV testing and counseling • Referral to ART and PrEP services	• Dispense medicines through the clinic	Both facility-based delivery via Mulago Hospital and satellite sites, and community-based via drop-in centers, and outreach activities in informal areas where PWUD congregate
Hope and Beyond Rehabilitation Center	Rehabilitation center for alcohol and substance use disorders	• Since 2012, has served approx. 590 clients^4^	• Referral to Butabika MAT clinic for MOUD • Psychosocial support	• Clients undergo HIV testing prior to admittance • Referral for HIV services, e.g., ART	• Yes	Facility-based delivery via the rehabilitation center
Serenity Rehabilitation Center	Faith-based rehabilitation center for alcohol and substance use disorders	• 30 bed-capacity^5^	• Psychosocial support	• Condoms are not part of the services offered	• Yes	Facility-based delivery via the rehabilitation center

^1^Butabika National Referral Mental Hospital (n.d.), *About Butabika National Referral Mental Hospital*. Retrieved 29 January 2026 from https://www.butabikahospital.go.ug/about-us/history-of-butabika-hospital

^2^Uganda Harm Reduction Network (2019). *Uganda Harm Reduction Network Annual Report 2019*. Retrieved 29 January 2026 from https://ugandaharmreduction.org/organization-documents/

^3^UNFPA. (10 October 2018). *Reaching out to most-at-risk populations: No one should be left behind in the battle against HIV*. Retrieved 29 January 2026 from https://uganda.unfpa.org/en/news/reaching-out-most-risk-populations-no-one-should-be-left-behind-battle-against-hiv

^4^Hope and Beyond (2022). *Hope and Beyond Report 2020–2022.* Retrieved 29 January 2026 from https://www.hopeandbeyondug.org/

^5^Registered and Licensed Health Units in Uganda. *Serenity Medical Centre*. Retrieved 29 January 2026 from https://uga.databasesets.com/registered-and-licensed-health-units-in-uganda/guid/0512

**Table 2:** Demographic characteristics of healthcare workers who completed an in-depth interview (*N* = 28) at 5 program sites, Kampala, Uganda

Demographic Characteristic of Healthcare Workers (*N* = 28)	*N* (%) or Median [IQR]
Program sites	
Butabika National Referral Hospital MAT Clinic	6 (21)
MARPI Clinic	6 (21)
Harm Reduction Network	6 (21)
Serenity Center	5 (18)
Hope and Beyond	5 (18)
Sex	
Male	15 (54)
Female	13 (46)
Age (years)	32 [28, 36]
Highest level of education	
University/college	27 (96)
Secondary	1 (4)
Healthcare Provider Classification^1^	
Social Worker	8 (29)
Psychologist	7 (25)
Counselor	5 (18)
Nurse/Nurse Counselor/Psychiatric Nurse	8 (29)
Clinical Officer^2^	3 (11)
Doctor	1 (4)
Other^3^	5 (18)
No. of years at current clinic	3 [1, 6]
No. of years experience providing HIV care	5 [2, 6]
No. of years experience caring for PWUD	4 [2, 6]

^1^More than one health cadre option may be selected; ^2^Defined as mid-level providers (similar to physician assistants in the U.S. context); ^3^Other category includes administrator, community health worker, community linkage and referral peer, occupational therapist, and project coordinator; Abbreviations: PWUD=people who use drugs

**Fig. 1:**
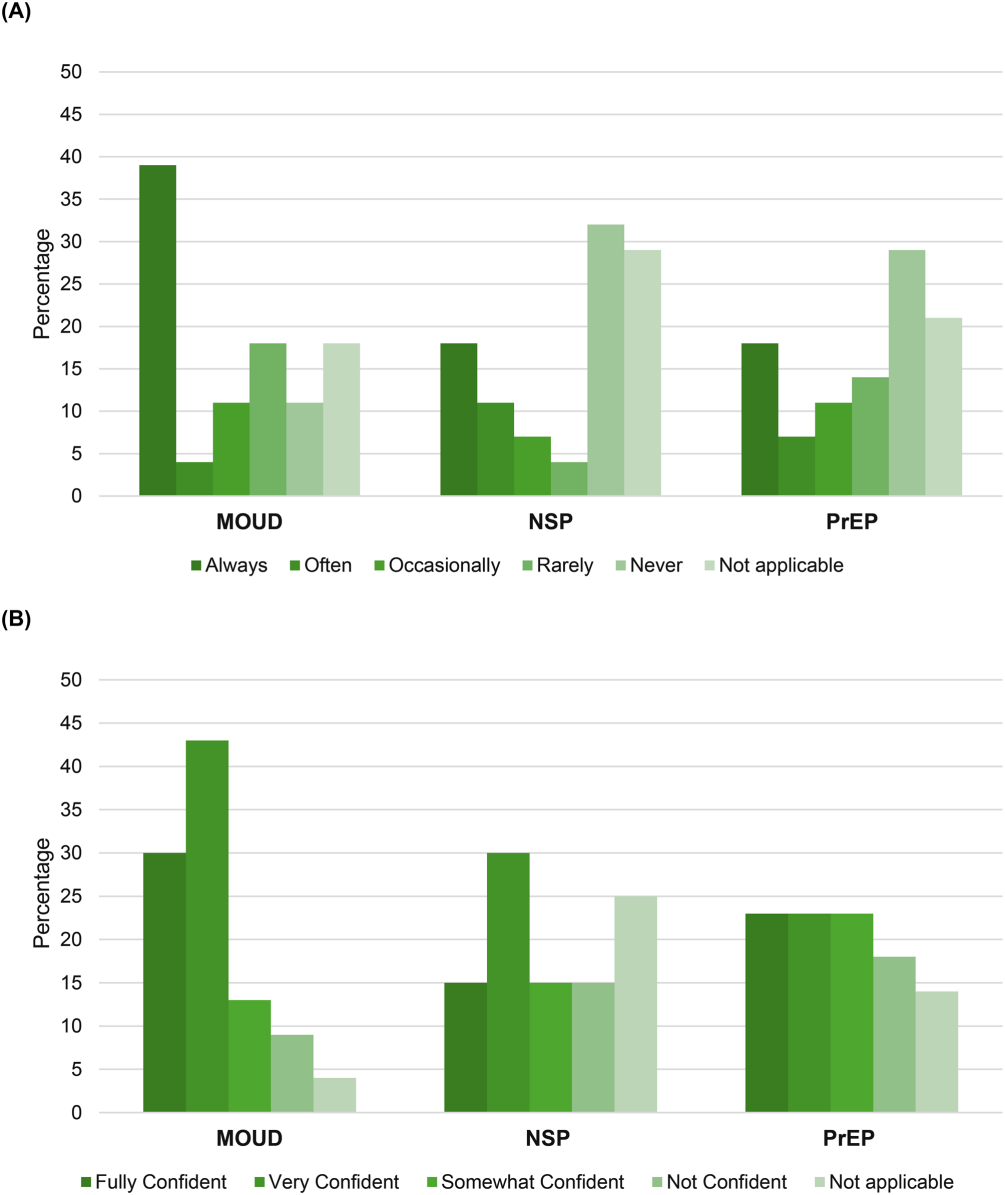
Healthcare worker experience and level of confidence with MOUD, NSP, and PrEP (**A**) Based on your role, how often are you involved in providing MOUD, NSP, and PrEP for people who use drugs (n = 28); (**B**) What is your current level of confidence in providing MOUD (n = 23), NSP (n = 20), and PrEP (n = 22) for people who use drugs (Excludes HCWs who responded ‘Not applicable” in Fig. 1**A**); MOUD=medications for opioid use disorder; NSP=needle and syringe exchange program; PrEP = pre-exposure prophylaxis

Based on their specific program site (i.e., MOUD, NSP, or rehabilitation center), we found that participants had varied levels of experience and confidence providing MOUD, NSP and PrEP services (Fig. 1). This was expected given the differing focuses of the programs, participants from programs that provided those services reported more familiarity and confidence. We did not observe differences in perspective across HCW roles. Overall HCWs believed that PrEP integration was highly acceptable and shared that PrEP integration was appropriate for and would benefit the population they served. However, HCWs were concerned that integrated service delivery could be challenging to implement in practice, and recommended strategies that could improve the process of PrEP integration, including decentralized services, peer engagement, provider training, policies that support expanded PrEP access, and community sensitization.

### Acceptability

We found that regardless of service delivery organization, PrEP integration into MOUD and NSP service programs was highly acceptable to HCWs providing treatment and care to PWUD; 89% strongly agreed that they liked the idea (Table 3). Although one participant thought PrEP integration was beyond the scope of services being offered at their specific site (i.e., rehabilitation center), overall, HCWs were enthusiastic about providing PrEP through MOUD and NSP programs because “they are ‘sisters,’ they walk together” and this integrated approach would allow clients to access a “whole package” of services (Intervention Characteristics: Relative advantage).

**Table 3:**
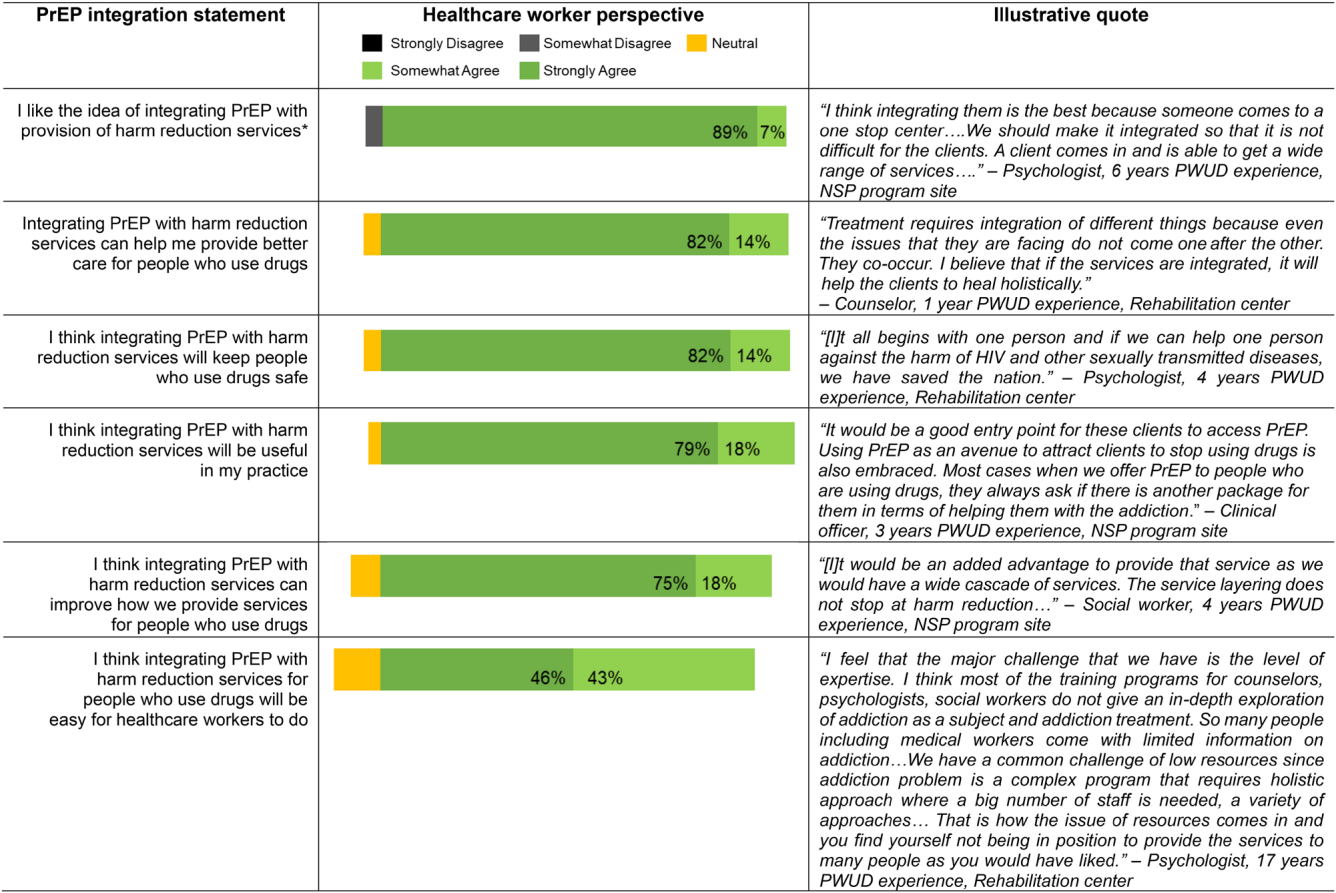
Healthcare worker perspectives on oral PrEP integration into MOUD and NSP services for people who use drugs

^1^Perceptions were measured using a Likert scale from 1 to 5 (1 = strong disagreement; 5 = strong agreement); for ease of interpretation, “strongly agree” appears before “somewhat agree” in the bar chart display. *All questions were drawn directly from the survey; but in conversation, harm reduction defined as MOUD and NSP services

*[I]t will be like a full course meal*,* because if it was one*,* if you give them only PrEP and not the needle*,* PrEP some people do forget*,* especially when they are high*,* they can forget to swallow their pill so it will be better if we give them both*,* the needle*,* and the PrEP…In case he forgets*,* he has not shared the needle with another person. I think it will be better if it is the whole package*,* provided at once. – Social worker*,* 5 years PWUD experience*,* NSP program site*.

HCWs attitudes about integration of PrEP with MOUD and NSP services demonstrated the high priority they placed on implementing strategies that provided access to HIV prevention for this key population (Inner Setting: Relative priority).

*What we need to keep in mind is that at least PrEP can prevent HIV and that is one thing that we need to have….It is one of the services that we need to have because we cannot stop the behavior but we can stop HIV. – Social worker*,* 4 years PWUD experience*,* NSP program site*

*If there is anything that can save our clients from acquiring HIV*,* we are ready to serve the clients. I think it is very important to offer HIV prevention services particularly PrEP because it will help us achieve our main goal of saving lives. – Counselor*,* 1 year PWUD experience*,* Rehabilitation center*

### Appropriateness

Regardless of program site, HCWs perceived that PrEP was beneficial for their client population and met their needs. Participants acknowledged that while under the influence, PWUD engaged in behaviors that increased their potential exposure to HIV, such as having sex without a condom, having sex with someone of unknown HIV status, or sharing injection equipment (Outer Setting: Patient needs and resources).

*[Y]ou know that some of the activities that they do can expose them to HIV….most of our clients are people who inject drugs*,* they use heroin. They share syringes as they inject the heroin in the ghettos*,* some people do not know where those syringes are coming from. They can get a syringe to use when they do not know that someone has already used it*,* when they do not know that person’s serostatus. I think if we initiate the PrEP services*,* it will help them a lot. – Social worker*,* 9 months PWUD experience*,* MOUD program site*

HCWs believed that being able to access PrEP through MOUD and NSP programs provided a relative advantage to providing these services separately via HIV clinics within public health facilities. When asked whether they thought integration would help them provide better care to PWUD, would keep PWUD safe, whether PrEP integration would be useful in their practice, and would improve how they provided services for PWUD, the majority of HCWs strongly agreed with these statements, regardless of program site (82%, 82%, 79% and 75%, respectively; Table 3).

HCW recognized that adding PrEP to the menu of prevention services available at facilities that focused on delivering services to PWUD offered an entry point for them to access these services. PWUD engagement with the healthcare system provided HCWs additional opportunities to counsel about risk behaviors, educate about PrEP benefits, and potentially reduced the stigma experienced when accessing services at facilities that provided care to the general population (Intervention Characteristic: Relative advantage).

*I think it would be a good idea to incorporate all services in one place because the drug users are not accepted in other facilities because people have the perception that they still use drugs. If the services they need are all in one place*,* they will feel comfortable receiving all the services from one place….because this is their safe place. – Social worker*,* 6 years PWUD experience*,* MOUD program site*

Furthermore, HCWs perceived that integrating services addressed other known client barriers to health service utilization, such as long waiting times experienced at facilities, challenges with loss to follow-up when PWUD were referred, and lack of funds for transportation if PWUD had to travel to different locations to access other services. In this way, integration was viewed as a strategy to improve overall utilization of prevention services for PWUD because they could conveniently access all the services they needed in a “one stop center” (Intervention Characteristics: Relative advantage).

*That is what I was talking about*,* clients will feel comfortable*,* and as you know that chain of referral can make someone change their mind should I go or shouldn’t I*,* and you know*,* one can end up giving up because if we do not have PrEP here*,* we refer them to other facilities but if it is here I feel like it would be easier and of great impact compared to how it is currently. – Social worker*,* 2 years PWUD experience*,* NSP Program site*

### Feasibility

Although HCWs perceived PrEP integration as highly acceptable and appropriate for their client population, they were less certain about its feasibility. When asked if they thought PrEP integration would be feasible, 89% agreed, but only 46% strongly agreed with this statement. Although HCWs believed that integrating PrEP was a good idea, they were concerned that it might be challenging to implement in practice (Table 3), especially within community-based service delivery settings. HCWs recognized that, depending on the substance use site and services offered, PrEP integration may not be compatible with existing workflows.

*[W]e are into addiction management then you are bringing in PrEP for HIV*,* they will be quite parallel.…if we are into addiction management*,* then we are going into HIV prevention which may create some friction within our own niche. We have not been into HIV and specifically HIV management or prevention. But I am sure we can still integrate it though it will be a step-by-step process. – Occupational therapist*,* 3 years PWUD experience*,* Rehabilitation center*

Resource constraints, including insufficient funding, trained staff, medications, and supplies, further restrict the ability to provide integrated care for PWUD. Furthermore, some sites may incur more costs than others when integrating PrEP, depending on their existing infrastructure for delivering services (Inner Setting: Compatibility).

*As a facility*,* if it is already established*,* you would incur a few costs in line with training people*,* the space is available. The required resources would go into mobilization*,* training health workers or mentorships and probably support supervision to see how the program is being run. It would not be costly in an established facility. – Social worker*,* 4 years PWUD experience*,* NSP program site*

In addition to material, financial, and human resources needed to ensure the feasibility of PrEP integration, across sites, HCWs highlighted the importance of resource continuity, such as continuous access to medications to sustain service delivery and support ongoing implementation at scale without interruption or delay (Characteristics of Systems: Resource continuity).

*I think that if we are to provide integrated PrEP services here at the MAT clinic*,* they need to give us enough medication that we will need because our clients do not have patience in them…….we just have to make sure that the medications are always there! A client will come … when he wants his medication*,* and then you start saying “let me first go and check in the store if it is there*,*” at that point the client will say “anyway*,* health worker (nurse/doctor) I will come back tomorrow”… another will say “but the boda-boda is leaving me” so it is better if we make sure that everything is there in place whenever the client needs it*. *– Social worker*,* 9 months PWUD experience*,* MOUD program site*

### Recommendations for PrEP integration

Based on their experiences providing services to PWUD, majority of HCWs were enthusiastic about PrEP integration and recommended several strategies to support successful implementation. These strategies included peer-led community-based service delivery, community sensitization, advocating for supportive policies to expand PrEP access, and building healthcare worker capacity to deliver integrated services.

*It feels good giving someone a service they need; it is satisfying because at the end of the day when they add the service it is our clients that will benefit. – Nurse counselor/ Psychiatric nurse*,* 3 years PWUD experience*,* MOUD program site*

### Decentralize services

HCWs felt that expanding access to PrEP, both knowledge and the medicines themselves, required placing services closer to where PWUD live. As a result, HCWs were most enthusiastic about decentralizing services because community-based PrEP integration strategies would “*take services to clients where they are”* and address barriers to service utilization.

*If you talk about PrEP in the perspective of drug use*,* access to PrEP is somehow complicated because you realize that not every pocket of our population can access PrEP or can easily access these PrEP services or even information concerning PrEP so there is still a big gap as far as access to services is concerned. The reason is that most of the services are centralized and one of the most effective approaches to delivering HIV services to [the] PWUD community should be more of through community initiatives like the outreaches like the peer-to-peer approaches*,* mobile clinics*,* which aim at reaching the community. – Social worker*,* 6 years PWUD experience*,* NSP program site*

### Utilize peer-to-peer strategies

HCWs suggested that peers could be utilized in both facility- and community-based service delivery because they possess knowledge of how to effectively reach PWUD and are accepted by the community. HCWs were especially excited about engaging peers for home deliveries (or community refills) of medications, which also addresses known barriers to service utilization, such as, transportation challenges and stigma.

*[W]e have tried to strengthen the community arm so you find that we employ the peer model which is supporting so much in the community. So you find in circumstances where there are limited resources since our peers are very resourceful and we can engage them. So with the little resources*,* we can sustain the community service since they can support things like commodity distribution; HIV self-testing kits*,* needle syringes*,* and even pick out the used ones. So through the community arm with the support of our peers*,* we ably try to resolve most of these problems. – Nurse/Nurse Counselor*,* 5 years PWUD experience*,* NSP program site*

In addition to physically delivering medications, HCWs viewed peers as health information resources to ensure that PWUD received accurate information about services available through the programs, as well as dispel any misinformation that may exist in the community. Furthermore, peers were considered a mechanism to support linkage and engagement with facility-based services. HCWs believed that engaging peers in the clinics was a strategy that could address stigma-related barriers and create a more “friendly” environment that would improve service utilization.

*They saw that we had people [peers] who valued them*,* who were not judging them*,* they felt safe and that is why having a safe space for people who use drugs really encourage them to access services – Social worker*,* 2 years PWUD experience*,* NSP program site*

### Build healthcare worker capacity

From the interviews, participants who expressed low confidence about PrEP felt they needed more knowledge to improve their ability to provide PrEP. One participant shared that they would be able to provide PrEP to their clients if they knew how to screen and determine PrEP eligibility and had information about PrEP to share with their clients. Similarly, access to knowledge and information was the reason some HCWs expressed less confidence in their abilities; at the time of the interview, some had not yet participated in MOUD or NSP training, as relevant to their role. Therefore, HCWs noted the importance of factoring in training to build competency and confidence in delivering PrEP, MOUD, and NSP services, and to improve readiness for implementing PrEP integration, especially among non-clinician HCWs.

*[C]ontinuous capacity building is needed because service provision is dynamic and not static. Events change according to the needs and desires of the community. This means that we have to work within what can make their lives better and keep them safe and reduce damage*. *– Clinical officer*,* 6 years PWUD experience*,* NSP program site*

Other factors that HCWs felt would create a supportive environment included additional personnel, training, and material resources such as job aids to support HCWs and improve their ability to tailor services to meet the unique needs of their clients.

*I would also feel that there is need for continuous training for even myself and other providers like me who are behind these interventions because obviously learning does not end and there is a need to upgrade due to the new dynamics and times. – Social worker*,* 6 years PWUD experience*,* NSP program site*

HCWs acknowledged the contribution of their own values and attitudes to PWUD experiences while accessing healthcare services, noting how negative experiences at health facilities, especially with providers, contributed to PWUDs’ preference for having services in the community or delivered by peers. Therefore, participants recommended creating clinic environments, *“such that there is friendliness towards harm reduction clients.”*

*I think the services that are available would be good for our community if we build up the friendly services within the facilities. As health workers we also have our beliefs*,* we have our values*,* so if we do not work on the values and beliefs that we health workers have then we shall not be able to serve the community*,* if we work on our values and beliefs then we shall be able to serve them. – Psychologist*,* 6 years PWUD experience*,* NSP program site*

Furthermore, HCWs believed that having “champions” to help encourage PrEP activities could help ensure HCWs remain knowledgeable and motivated to continue providing PrEP longer term. Champions would be knowledgeable about PrEP and provide encouragement and motivation to the larger HCW team. HCWs felt that this implementation “champion” should be a counselor, not a peer, even though a majority agreed that peers were important; the whole team works together.

*[W]e need a focal person….and given our community that focal person has to be a counselor rather than a peer because we have had substantial challenges around peers given the history of substance users… we have constant challenges like attending the MAT clinic where they [peers] sometimes come late or even do not come at all. Sometimes*,* the peers relapse….they are just like any other clients who use drugs.* – *Doctor*,* 1 year PWUD experience*,* MOUD program site*

### Expand PrEP access

Aligned with the goal of creating supportive environments that would increase service utilization among PWUD, HCWs mentioned the importance of having supportive policies to scale-up services, such as NSP, beyond current reach and to lower barriers to community-based delivery of services. For example, Uganda Harm Reduction Network’s drop-in-centers are not allowed to initiate ART or PrEP because the policy framework does not currently exist.

*I think it is a limitation within the guidelines and SOPs [standard operating procedures]. At our level we cannot enroll people on ART neither can we enroll them on PrEP and what we do at community level is monitor*,* offer support care to the client and even do follow up…. Even before we think about integration we should have a supportive policy framework.…we need to have DICs [drop-in-centers] accredited and once they are accredited then they get to a level when they can be given a green light to offer that comprehensive care. It starts from that end… – Social worker*,* 6 years PWUD experience*,* NSP program site*

HCWs also felt that guidelines should support access to new PrEP formulations, such as long-acting injectable formulations, that might reduce stigma and therefore increase uptake. Longer-acting PrEP products were viewed as advantageous because they would reduce visit frequency, thereby addressing transportation challenges, and reducing stigma, as the innovation would be packaged differently from antiretrovirals.

*…the packaging is almost the same as ARVs*,* by the way*,* these are ARVs*,* though their contents differ. It is TDF [tenofovir disoproxil fumarate]. And some say if my partner sees me with this (PrEP)*,* they will think I am not negative…and some just do not want to take their daily pill. They will prefer if it was an injection*,* they inject like 2 or 3 months and that would be better*,* that is what they say. – Social worker*,* 5 years PWUD experience*,* NSP program site*

### Community sensitization

HCWs believed that sensitization about PWUD was essential to successful implementation of PrEP into MOUD and NSP services.

*One is stigma in the community*,* the community still views the drug practice as immoral and against the values of the community so this continues to affect people who are using the drugs to do their activities underground. – Social worker*,* 4 years PWUD experience*,* NSP program site*

Providers recommended that, regardless of who is involved, the sensitization process needs to begin with understanding addiction and education about addiction and substance use services for PWUD.

*[A]ny stakeholder*,* any organization that wants to integrate PrEP with harm reduction*,* they should first know about addiction because handling people who use drugs is not an easy thing. Some people always mistake*,* thinking that the programs that work in HIV care can be done in the same way with people who use drugs…. I think before they integrate HIV and harm reduction they should really know about addiction*,* and the nature and the category of PWUD before putting in place any PrEP SOPs [standard operating procedures]. – Social worker*,* 9 months PWUD experience*,* MOUD program site*

Community sensitization was perceived as a continuous ongoing process because the way that addiction is understood in the community affects PWUD patterns of health service utilization as well as their experiences while accessing these services.

*Many people*,* especially here in Uganda*,* do not understand this as a disease which leads to a lot of judgement for the clients and most times when the family members come in for family sessions*,* they continuously judge them since they believe it is a behavioral issue yet it is a disease that these clients are suffering from. – Counselor*,* 1 year PWUD experience*,* Rehabilitation center*

HCWs advocated for clear messaging about PrEP in order to combat community stigma. They believed that PrEP advocacy was necessary to change knowledge and attitudes about PrEP and generate demand for it in the community.

*We need proper advocacy to the lay man so that they know that there is this treatment that can prevent them from acquiring HIV. This is good because even when the relatives get to know that so and so is on PrEP*,* they can understand. This will greatly improve on the retention because you can preach to a client however*,* when family gets to know that so and so is taking PrEP*,* it can affect their relationship greatly. – Doctor*,* 1 year PWUD experience*,* MOUD program site*

## Discussion

In this formative qualitative evaluation, we identified HCWs’ perceptions about the acceptability, appropriateness, and feasibility of PrEP integration with MOUD and NSP service programs in Kampala, Uganda. Our study identified that PrEP, MOUD and NSP expertise varied by program site and were siloed; HCWs were either experienced with substance use services or experienced with PrEP but did not have the competency or confidence to perform tasks that were beyond the scope of their primary responsibilities. Therefore, HCWs across program sites would benefit from capacity building activities that include cross-training to expand their abilities to provide both PrEP and substance use services and improve readiness for PrEP integration. Although not focused on PWUD and PrEP integration with MOUD and NSP services, other studies have demonstrated the benefits of cross-training to expand knowledge and skills, mitigate staffing shortages, and improve job satisfaction [47, 48].

We found that HCW perception of the acceptability and appropriateness of service integration was informed by their experiences providing substance use services and/or PrEP to PWUD. They recognized that PWUD would benefit from HIV prevention services, and as a result, believed that PrEP integration with MOUD and NSP services met client needs and addressed barriers to service utilization, such as stigma and transport challenges. Strategies that would optimize PrEP integration for PWUD include the availability of longer-acting injectable PrEP products [49] and community-based refill models [26, 27], that would also need to integrate laboratory services so that data are available to inform care. A similar approach was used to expand PrEP access through pharmacies, which are often the first point of entry for individuals seeking healthcare [50, 51]. In Kenya, pharmacy-based PrEP integration models have been successful in expanding access and increasing PrEP uptake among AGYW [52, 53] and the general population [54].

A key finding from our study was that HCWs were supportive of utilizing peers in service delivery approaches, which is aligned with other studies that have used peers to deliver services to PWUD [5, 28] and other populations [29–33]. In a study conducted in Kampala, Uganda, peer educators were trained to assist with recruitment and mobilization [5]. The involvement of peer educators alleviated participant concerns about confidentiality and fears regarding legal repercussions due to study participation [5]. In a previous qualitative evaluation focused on understanding PWID experiences with MOUD, NSP, and PrEP services [55], our team reported that PWID were highly influenced by their peers and that their willingness to take PrEP was based on information they received from peers. Therefore, utilizing peers would not only increase reach and engagement but also increase access to health services for PWUD, as peers can directly bring services to individuals in the community, while making clinic spaces feel friendly and accepting.

A comprehensive approach that addresses HCWs’ capacity, along with community sensitization about addiction and PrEP, can enable successful PrEP integration into MOUD and NSP programs. Community perceptions and attitudes about addiction impact patterns of healthcare utilization [56, 57]. Therefore, prior to implementation, it is important to promote environments to support the overall well-being of PWUD using approaches that build knowledge about and compassion for those struggling with addiction, along with advocating for policies that remove barriers to community-based access to PrEP.

We found that HCWs were concerned about resource continuity. Ever more challenging in 2026, given changes to the U.S. government priorities and foreign aid, it is still essential to ensure that evidence-based interventions, such as PrEP, MOUD, and NSP services, are sustained and accessible to PWUD, through consistent product availability is essential. Availability of resources and supplies is a key determinant of whether individuals seek and use services, as shortages or limited supply can reduce both accessibility and quality of service delivery [58, 59]. In addition, resource scarcity and uneven distribution of services influence the adoption and utilization of health services [60].

Our analysis had several strengths. The five participating program sites represented diverse settings that provide services to PWUD, and therefore, HCWs’ perceptions of the acceptability, appropriateness, and feasibility of PrEP integration may be representative of the opinions of other HCWs providing care and treatment to this key population. An additional strength, we used the CFIR as a framework to guide development of the interview guide and our understanding of provider perceptions of PrEP integration with MOUD and NSP services during data analysis. Use of the CFIR framework in a low-middle-income country (LMIC) setting adds to the literature demonstrating relevance and application of this determinants’ framework in LMIC settings and supports comparison with studies conducted in similar settings. Our analysis also had limitations. Although our program sites represented diverse settings, all sites were in Kampala, and as a result, the strategies recommended by HCWs to support integration may not be generalizable to other facilities or geographic regions. Furthermore, because programs supporting MOUD and NSP services are limited, we also included HCW perspectives from in-patient and out-patient rehabilitation centers, which we acknowledge have different organizational cultures from MOUD and NSP program sites.

## Conclusions

Integration of PrEP with MOUD and NSP service programs was perceived by HCWs as acceptable, appropriate, and feasible to implement for people who use or inject drugs. Achieving impact will require leveraging strategies that optimize available resources, such as the peer delivery model, which may address challenges related to resource continuity and program sustainability in efforts to end the HIV epidemic.

## Supplementary Information

Below is the link to the electronic supplementary material.

Supplementary Material 1

## Data Availability

Data cannot be shared publicly because of the sensitive nature of qualitative data. The data are available upon reasonable request for researchers who meet criteria for access to qualitative data.

## Abbreviations

ART: Anti-retroviral therapy
CFIR: Consolidated framework for implementation research
DIC: Drop-in center
HCW: Healthcare workers
PrEP: Pre-exposure prophylaxis
PWID: People who inject drugs
PWUD: People who use drugs
MARPI: Most-at-risk population initiative
MOUD: Medications for opioid use disorders
NSP: Needle and syringe exchange programs
UHRN: Uganda harm reduction network
