# Supplementary material for: “[I]t will be like a full course meal”: healthcare worker perspectives on strategies to optimize integration of PrEP into medication for opioid use disorder and needle and syringe exchange programs for people who use drugs in Kampala, Uganda
Source: Addict Sci Clin Pract. 2026 Jun 27;21:53. doi: 10.1186/s13722-026-00693-z (PMC13310434; doi:10.1186/s13722-026-00693-z)
Supplement: Supplementary file 1 — Supplementary Material 1 [file 13722_2026_693_MOESM1_ESM.docx]

**Supplementary Material: Semi-structured interview guide to elicit healthcare worker perspectives on PrEP integration with harm reduction services in Kampala, Uganda**

**Guide details:**

Version 1.1

June 29, 2021

**Interviewer Instructions:**

The following is a guide. Try to ask all the questions below in the order given, but it is more important to maintain the flow of discussion. Suggested probes have been included.

******************************************************************************************************************************

**Before turning on the recorder, start with the following introductory script:**

*Hi, my name is ____________. Thank you for agreeing to participate in an interview today. As you might know, drug trafficking and drug use has risen in Uganda over the past decade. People who use drugs are at increased risk of getting HIV. Programs that help reduce harm among those who use drugs, and simultaneously prevent HIV, could help make sure people who use drugs stay as safe as they can and also avoid getting HIV. We would like to talk with you today about how we can best design programs that reduce harm and prevent HIV among people who use.*

*We are interested in all your thoughts and ideas about these programs. During our discussion today, I will ask you questions that you are free to answer in any way you wish. Feel free to elaborate on any of your points. If a question is unclear to you, please feel free to ask me to explain it.*

*I would like to record the discussion so I don’t miss anything that you say. I will not include your names on any documents or in the recording. Your responses will be kept confidential, which means we will keep what you say private from others. Is it okay if I record our discussion?* [Wait for the participant to give verbal consent to recording]

*Before we start I would like to remind you that there are no wrong answers during our discussion. We are interested in knowing what you think, so please feel free to be open and share your point of view. We hope you can help us understand how to best design these programs. Your comments about what will not work are just as helpful as your comments about you think will work.*

*If you want to stop the discussion at any time, just let me know. Do you have any questions for me before we get started? [Wait for participant to respond – answer any questions they have]*

*I am turning on the recorder now.*

******************************************************************************************************************************

**Before beginning interview questions, please read the following script for the recording:**

*Today is [day of week], [month, day, year] and it is now [time of day]. This is interview [ID number] conducted at [discussion location].*

**You are now ready to begin asking the questions outlined below.**

1. First, I’d like to learn a little more about your clinic/facility, including how things work now, and what the overall climate is like.
   - What kinds of challenges do you routinely face as a team and how did you resolve them? *[Inner Setting: Networks and Communication]*
   - Do you feel like you have the capacity to change the way things are done at your clinic/facility? Why or why not? *[Inner setting: implementation climate: learning climate]*
   - What are the new things that you try to improve your work processes? *[Inner setting: implementation climate: learning climate]*
   - Do you feel like you have the time and motivation to think about ways to improve things generally in your facility?
   - To what extent are new ideas embraced and used to make improvements in your organization? Can you describe a recent example? *[Inner Setting: Culture]*
2. Now, I would like to learn a little bit more about what you know about harm reduction services for PWUD.

- What do you know about harm reduction (MAT or NSP) for people who use drugs? *[Characteristics of Individual: knowledge and belief about the intervention]*
- How do you feel about your ability to provide harm reduction services (MAT or NSP) for people who use drugs? *[Characteristics of Individuals: Self-efficacy]*

1. Now, I would like to learn a little bit more about what you know about PrEP for HIV prevention.

- What do you know about HIV prevention with oral PrEP? *[Characteristics of Individual: knowledge and belief about the intervention]*
- How do you feel about your ability to provide oral PrEP for people who are at risk for HIV? *[Characteristics of Individuals: Self-efficacy]*
- Can you tell me about particular characteristics of people who use drugs that would make them more or less of a priority population for oral PrEP or other HIV prevention services? *[Characteristics of Individual: knowledge and belief about the intervention]*

1. Now I’d like to learn more about how you think about HIV prevention for PWUD.
   - Overall, how do people feel about the current programs/practices/processes that are available for PWUD? Are they working? Why or why not? *[Inner Setting: Implementation Climate – tension for change]*
   - Thinking about health care services for PWUD, what do you think PWUD need most? Where would you rank HIV prevention/harm reduction and addiction care? *[Inner setting: implementation climate: relative priority]*
   - How do you think providing integrated PrEP and needle exchange or methadone services compares to providing these services separately? *[Intervention Characteristics: Relative Advantage]*
   - What additional information do you think would be needed to get your clinic/facility on board with implementing HIV prevention services, including harm reduction services for PWUD? *[Intervention Characteristics: Strength of Evidence]*
   - How well does providing HIV prevention services and harm reduction services for PWUD fit with the objectives of your facility/clinic/program? *[Inner Setting: Compatibility]*
2. Based on what you know about providing harm reduction services (NSP and MAT) for PWUD or/and providing oral PrEP*, I’d like to learn more about how you think they could be provided together as services for PWUD.
   - How might adding oral PrEP to existing services for PWUD meet the needs and preferences of PWUD? How might it not meet their needs and preferences? *[Outer setting: patient needs and preferences]*
   - How might these services be altered or organized to better meet their needs and preferences?
   - What barriers do you think the people you serve will face with accessing oral PrEP services at your facility?
   - What barriers do you think the people you serve will face with accessing NSP/MAT services at your facility?
3. Now I’d like you to think about what it would look like if you were to move forward with delivering oral PrEP alongside other services for PWUD at your facility/clinic.
   - What do you think will be the most challenging aspect of integrating oral PrEP delivery with MAT/NSP services? *[Intervention Characteristics: Complexity/Adaptability]*
   - Adherence to PrEP can be challenging. What are some of the ways this program can support adherence?
   - What kinds of infrastructure changes do you think you would need to make to accommodate providing integrated oral PrEP and NSP/MAT? This can include things like staffing changes and the physical layout of the clinic. *[Inner Setting: Structural Characteristics]*
   - What other resources do you think would be needed in order to provide integrated oral PrEP services and NSP/MAT services at your clinic/facility? *[Inner Setting: Readiness for Implementation]*
   - Who should we engage in order to provide integrated oral PrEP services and NSP/MAT services at your facility? Probe about: community leaders, facility in charge, program directors, implementing partners, church leaders *[Inner Setting: Readiness for Implementation]*
   - What costs do you think your clinic/facility will incur if you decided to implement oral PrEP services integrated with NSP/MAT services? *[Intervention Characteristics: Cost]*
   - What kinds of incentives might help ensure that the implementation of integrated services is successful? *[Inner Setting: Organizational Incentives and rewards]*
   - What training would need to take place before implementing integrated services? *[Inner Setting: Access to knowledge and information]*
   - What do you think your fellow staff would think about providing PrEP as another service that they have for PWUD?

1. Finally, I’d like to think about how you might go about implementing integrated oral PrEP and NSP/MAT services at your facility/clinic.
   - Do you know any providers who are providing exemplary services for PWUD? What makes them a good service provider? [Process: Reflecting and evaluating]
   - Tell me about any programs that are well-liked by PWUD? What makes these programs well-liked? Why do you say so[Process: Reflecting and evaluating]
   - Tell me about any programs that are now well liked by PWUD. Why do you think they are not liked*?* [Process: Reflecting and evaluating]
   - What would be the most important things to keep in mind when moving forward with programs that combine HIV prevention and harm reduction for PWUD at your facility? *[Process: Planning]*
   - Who would be the key influential people to get on board, both at the clinic and outside the clinic to help implement integrated oral PrEP and NSP/MAT services for PWUD at your facility? *[Process: Engaging]*
2. Now I would like to hear more about how COVID-19 has affected the services you are providing to PWUD.
   - How has the way harm reduction services delivered at your facility changed since corona virus?Probe about how appointment schedules changed, etc.
     - What happened with access to NSP and MAT during the corona virus?
     - What are some of the challenges your clients are facing with accessing harm reduction services these days?
   - How has the way HIV prevention services delivered at your facility changed since corona virus?
     - What has happened with access to oral PrEP? (for those that are offering PrEP)

Probe about concerns about resupply, drug shortages and stock outs during the nationwide shutdown.

- - - What are some of the challenges your clients are facing with HIV prevention these days?
  - In your opinion, how important is harm reduction/HIV prevention to your clients as compared to concerns about the corona virus? Why do you say this?

As we finish taking today, are there any other questions we should be asking people like you to help us understand whether and how oral PrEP and harm reduction services can be implemented at your facility/clinic?

Is there anything about harm reduction services (MAT or NSP) or HIV prevention (oral PrEP) that you think is important to tell me, but I didn’t ask about?

That is all of the questions I have for you today. Thank you for your time. If you do not have any further questions or comments, I will now turn off the recorder*.*

******************************************************************************************************************************

*If providers are unaware of oral PrEP, please provide them with the following explanation: PrEP is the medication that someone who is HIV negative can take to reduce their risk of contracting HIV. If someone takes the medication every day, it has been shown to be very effective at protecting them from HIV even if they are exposed to the virus.
